# Usefulness and Limitations of Comprehensive Characterization of mRNA Splicing Profiles in the Definition of the Clinical Relevance of *BRCA1/2* Variants of Uncertain Significance

**DOI:** 10.3390/cancers11030295

**Published:** 2019-03-01

**Authors:** Elisa Gelli, Mara Colombo, Anna Maria Pinto, Giovanna De Vecchi, Claudia Foglia, Sara Amitrano, Valeria Morbidoni, Valentina Imperatore, Siranoush Manoukian, Margherita Baldassarri, Caterina Lo Rizzo, Lorenza Catania, Elisa Frullanti, Enrico Tagliafico, Laura Cortesi, Federica Spaggiari, Maria Antonietta Mencarelli, Eva Trevisson, Paolo Radice, Alessandra Renieri, Francesca Ariani

**Affiliations:** 1 Medical Genetics, University of Siena, 53100 Siena, Italy; elisa.gelli@student.unisi.it (E.G.); annamaria.pinto@dbm.unisi.it (A.M.P.); va.imperatore@gmail.com (V.I.); catania4@student.unisi.it (L.C.); elisa.frullanti@dbm.unisi.it (E.F.); francesca.ariani@unisi.it (F.A.); 2 Unit of Molecular Bases of Genetic Risk and Genetic Testing, Department of Research, Fondazione IRCCS Istituto Nazionale dei Tumori (INT), 20133 Milan, Italy; Mara.Colombo@istitutotumori.mi.it (M.C.); giovanna.devecchi@cogentech.it (G.D.V.); Claudia.Foglia@istitutotumori.mi.it (C.F.); Paolo.Radice@istitutotumori.mi.it (P.R.); 3 Genetica medica Azienda Ospedaliera Universitaria Senese, 53100 Siena, Italy; sara.amitrano@dbm.unisi.it (S.A.); margherita.baldassarri@dbm.unisi.it (M.B.); lorizzo2@unisi.it (C.L.R.); mariaantonietta.mencarelli@unisi.it (M.A.M.); 4 Department of Woman and Child Health, University of Padova, 35128 Padova, Italy; valeria.morbidoni@gmail.com (V.M.); eva.trevisson@unipd.it (E.T.); 5 Istituto di Ricerca Pediatrica, IRP, Città della Speranza, 35129 Padova, Italy; 6 Unit of Medical Genetics, Department of Medical Oncology and Hematology, Fondazione IRCCS Istituto Nazionale dei Tumori (INT), 20133 Milan, Italy; Siranoush.Manoukian@istitutotumori.mi.it; 7 Department of Medical and Surgical Science, Center for Genome Research, University of Modena and Reggio Emilia, 41125 Modena, Italy; enrico.tagliafico@unimore.it; 8 Department of Oncology and Haematology, Azienda Ospedaliero-Universitaria Policlinico, University of Modena and Reggio Emilia, 41124 Modena, Italy; cortesi.laura@aou.mo.it (L.C.); hbc@unimore.it (F.S.)

**Keywords:** splicing, BRCA, mRNA analysis, VUS

## Abstract

Highly penetrant variants of *BRCA1/2* genes are involved in hereditary predisposition to breast and ovarian cancer. The detection of pathogenic BRCA variants has a considerable clinical impact, allowing appropriate cancer-risk management. However, a major drawback is represented by the identification of variants of uncertain significance (VUS). Many VUS potentially affect mRNA splicing, making transcript analysis an essential step for the definition of their pathogenicity. Here, we characterize the impact on splicing of ten *BRCA1/2* variants. Aberrant splicing patterns were demonstrated for eight variants whose alternative transcripts were fully characterized. Different events were observed, including exon skipping, intron retention, and usage of de novo and cryptic splice sites. Transcripts with premature stop codons or in-frame loss of functionally important residues were generated. Partial/complete splicing effect and quantitative contribution of different isoforms were assessed, leading to variant classification according to Evidence-based Network for the Interpretation of Mutant Alleles (ENIGMA) consortium guidelines. Two variants could be classified as pathogenic and two as likely benign, while due to a partial splicing effect, six variants remained of uncertain significance. The association with an undefined tumor risk justifies caution in recommending aggressive risk-reduction treatments, but prevents the possibility of receiving personalized therapies with potential beneficial effect. This indicates the need for applying additional approaches for the analysis of variants resistant to classification by gene transcript analyses.

## 1. Introduction

Breast cancer (BC) represents a priority public health problem, being the most common cancer diagnosed in women worldwide and a leading cause of cancer deaths [1,2,3]. The most relevant predisposing factor is family history. The risk increases with the number of affected relatives and the age of cancer onset. Approximately 5–10% of BC cases are associated with a strong hereditary component and 15–25% of familial aggregations are due to mutations in the *BRCA1* (MIM# 113705) or *BRCA2* (MIM# 600185) genes [4,5,6,7]. Recent estimates have assessed that women who inherit a mutation in *BRCA1* have a chance of 72% (95% confidence interval (CI), 65–79%) of developing BC and of 44% (95% CI, 36–53%) of developing ovarian cancer (OC) in their lifetime. In *BRCA2* mutation carriers the percentages are similar for BC (44%; 95% CI, 36–53%), but lower for OC (17%; 95% CI, 11–25%) [8]. BRCA gene mutations may also predispose to the development of other cancers, such as endometrial, pancreatic, colorectal, gastric, and skin cancer [9,10]. Genetic testing is thus essential to identify at-risk individuals who can undergo paths aimed at ensuring early diagnosis and/or at reducing the risk of cancers, including prophylactic surgery (mastectomy and/or oophorectomy). Moreover, it has been demonstrated that patients with BRCA mutation and OC present a significantly more favorable prognosis and pharmacological sensitivity to treatment with inhibitors of the enzyme poly (ADP-ribose) polymerase (PARP) [11].

The use of BRCA mutation testing for purposes of therapeutic choices implies that genetic testing is easily accessible for all patients who could benefit and that results are available in a timeframe consistent with the clinical need. Next-generation sequencing (NGS) platforms, allowing the simultaneous analysis of multi-gene panels, have significantly decreased the time required for genetic testing, but a major critical issue for clinical application of results is still represented by variant interpretation. Despite the testing of hundreds of thousands of patients worldwide, there is a significant percentage (5–20%) of *BRCA1/2* sequence changes that are classified as variants of uncertain significance (VUS), i.e., alterations for which there is insufficient evidence of the effects on the function of the gene product and disease risk [12]. Variant classification has greatly improved through the research efforts of multidisciplinary international consortia such as the Evidence-based Network for the Interpretation of Mutant Alleles (ENIGMA; http://enigmaconsortium.org/), which has collected evidence on a large-scale basis, and through the application of multifactorial likelihood models that take into account tumor characteristics and family-based genetic data [13]. However, given the large size of the *BRCA1/2* genes, many VUS are unique to only one or few patients and the amount of data required for a reliable classification by multifactorial models is often lacking.

It has been previously demonstrated that many *BRCA1/2* variants affect mRNA and generate abnormal splicing products [14]. These variants are able to alter intronic splicing elements such as the 5’ and 3’ consensus sequences, the so-called “Cartegni consensus regions” [15], or exonic/intronic regulatory elements; they can also introduce de novo splice sites or lead to the activation of cryptic splice sites. The resulting aberrant transcript(s) can present partial/entire intronic retention or exon exclusion with production of a premature termination codon (PTC) in most cases. This evidence of a dysfunctional role for the identified variants can thus overcome the need for additional data required for multifactorial likelihood analysis. Different in silico tools have been developed to predict the consequences of intronic/exonic variants on normal mRNA splicing and select them for in vitro studies [16,17,18]. However, their predictions have been demonstrated to be highly reliable for variants located in Cartegni-consensus regions, but less accurate for variants lying in elements such as the exonic splicing enhancer (ESE) or branchpoints [19]. Laboratory tests, including transcript analysis and minigene assay, have been demonstrated to represent powerful tools to assess pathogenicity of potential spliceogenic variants and remain mandatory for the characterization of aberrant transcripts [14,20,21,22,23,24,25,26,27,28,29,30,31,32,33,34,35,36].

In the present study, we investigated the splicing impact of ten *BRCA1/2* variants never before characterized in patient mRNA: four at the nearly invariant GT/AG dinucleotides at 5’ and 3’ intron ends (c.7618-2A>G, c.7806-2A>G, c.8331+1G>A and c.8331+2T>C in *BRCA2*), five in the adjacent less conserved splicing regions (c.302-5T>C, c.441G>C and c.441+5A>G in *BRCA1*; c.68-5A>G and c.476-3C>A in *BRCA2*), and one outside the Cartegni consensus sequence (c.213-14C>G in *BRCA1*). Real-time quantitative PCR (qPCR) was employed to evaluate the relative contribution of the different observed mRNA isoforms to the total transcript pool. A possible incomplete (“leaky”) spliceogenic effect was investigated using exonic tag single nucleotide polymorphisms (SNPs) or minigene assays, allowing the final assessment of pathogenicity according to the current guidelines of the ENIGMA consortium [37].

## 2. Results

**Figure 1:**
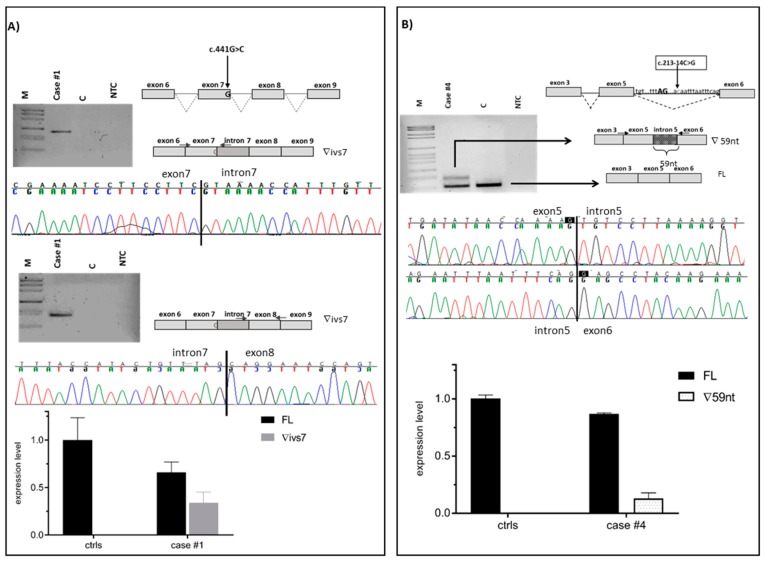
mRNA analysis of spliceogenic *BRCA1* variants. Amplified products were separated on 3% agarose gel and independently sequenced. Gel images, graphical representation, and exon–intron junction sequences of full-length (FL) and aberrant transcripts are shown in upper panels. Variants and RT-PCR primers locations are indicated by the arrows. qPCR results are shown as histograms displaying the relative expression of the full-length and aberrant transcript in the patient’s and controls’ samples (lower panels). (**A**) c.441G>C, causing the retention of intron 7 (∇ivs7). The aberrant transcript accounts for 0.34 (±0.11) of overall gene expression in the variant carrier (case #1) and is undetectable in controls. (**B**) c.213-14G>C, causing the retention of 59 nt at the 3′-end of intron 5 (∇59nt). The aberrant transcript accounts for 0.13 (±0.05) of overall gene expression in the variant carrier (case #4) and is undetectable in controls. M, molecular weight marker (0.15–2.1 Kbp, Roche); C, healthy control sample; NTC, no template control.

**Figure 2:**
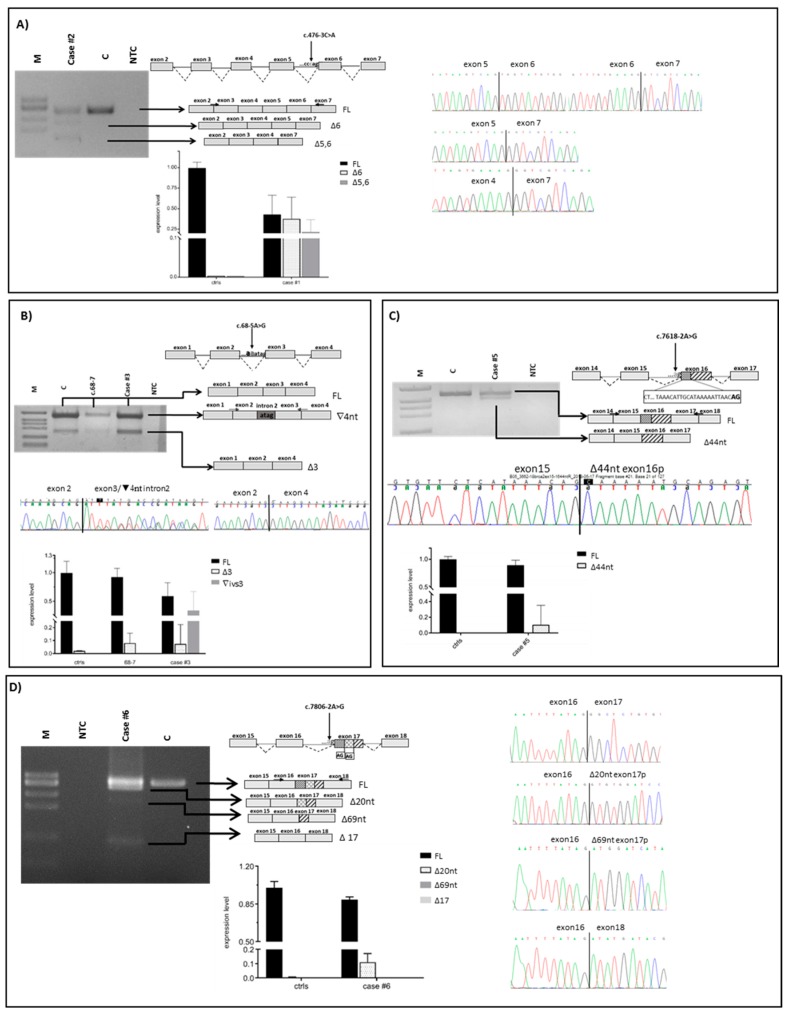

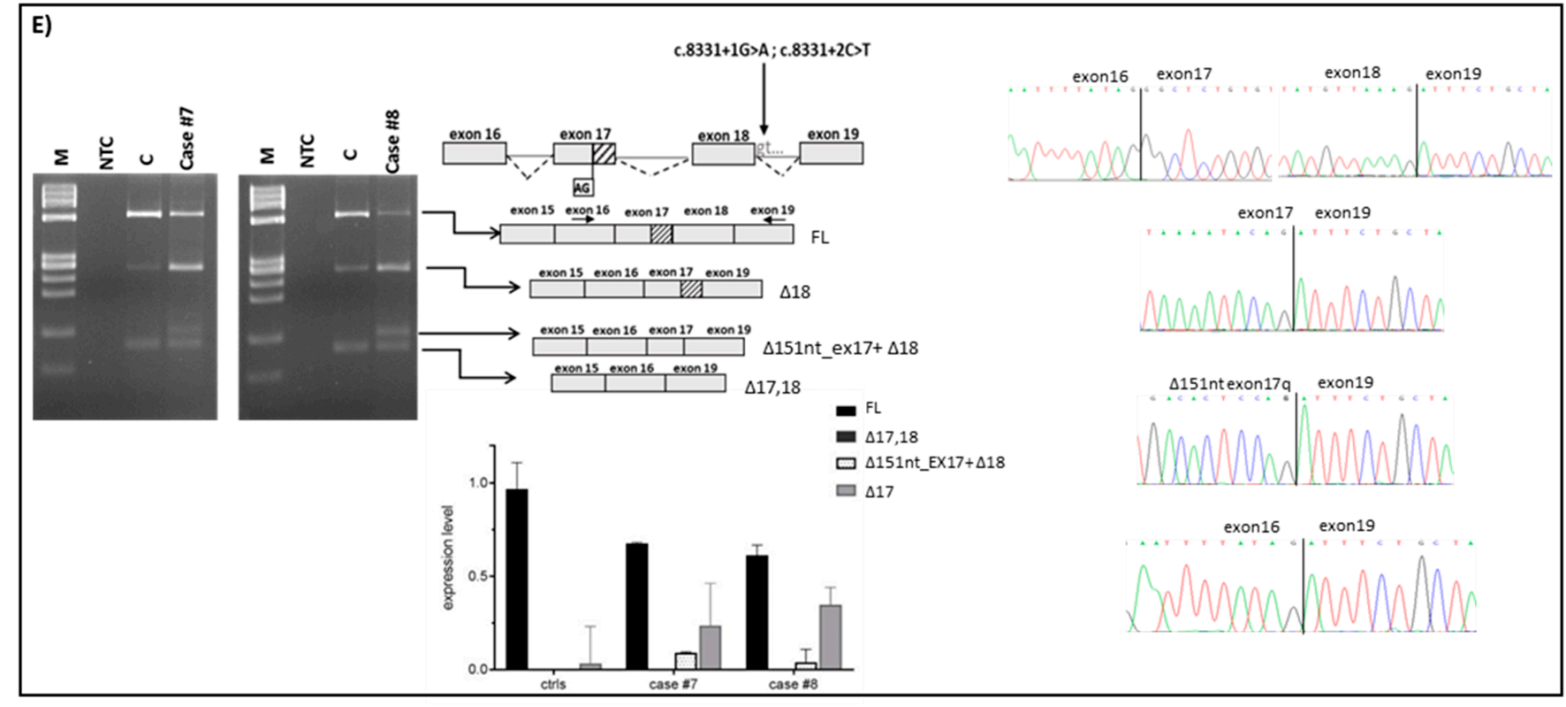
mRNA analysis of spliceogenic *BRCA2* variants. Amplified products were separated on 3% agarose gel and independently sequenced. Gel images, graphical representation, and exon–intron junction sequences of full-length (FL) and aberrant transcripts are shown in upper panels. Variants and RT-PCR primers locations are indicated by the arrows. qPCR results are shown as histograms displaying the relative expression of the full-length and aberrant transcript in the patient’s and controls’ samples (lower panels). (**A**) c.476-3C>A, causing the up-regulation of the isoforms lacking exon 6 (Δ6) and exons 5 and 6 (∆5,6). The Δ6 transcript accounts for 0.36 (±0.15) and Δ5,6 for 0.23 (±0.15) of overall gene expression in the variant carrier (case #2), and is nearly undetectable in controls. (**B**) c.68-5A>G variant, causing the retention of four nucleotide at the 3’-end of intron 2 (∇4nt) and the up-regulation of the isoform lacking exon3 (∆3). The aberrant transcript, detectable exclusively in the variant case (case #3), accounts for 0.34 (±0.3) of overall gene expression. The ∆3 isoform accounts for 0.07 (±0.24) of overall gene expression in the variant carrier, 0.08 (±0.08) in the c.68-7T>A sample, and 0.02 (±0.05) in controls. (**C**) c.7618-2A>G variant, causing the deletion of 44 nt at the 5’-end of exon 16 (∆44nt). The aberrant transcript accounts for 0.10 (±0.24) of overall gene expression in the variant carrier (case #5) and is undetectable in controls. (**D**) c.7806-2A>G, causing three different aberrant transcripts resulting from the deletion of 20 nt at the 5’end of exon 17 (Δ20nt), of 69 nt at the 5’end of exon 17 (Δ69nt) and of whole exon 17 (Δ17). Only the Δ20nt transcript is detectable in the variant carrier (case #6) and accounts for 0.11 (±0.06) of overall gene expression and is barely detected in control samples. (**E**) c.8331+1G>A and c.8331+2T>C variants, causing the deletion of 151 nt at the 3’-end of exon 17 and of the whole exon 18 (Δ151nt_ex17 + Δ18), and the up-regulation of the isoforms resulting from the deletion of exon 18 (Δ18) and of exons 17 and 18 (Δ17,18). The ∆151nt_ex17 + ∆18 transcript accounts for 0.09 (±0.001) and 0.04 (±0.06) of overall gene expression in c.8331+1G>A (case #7) and c.8331+2T>C (case #8) samples, respectively, and is undetectable in controls. The ∆18 transcript accounts for 0.23 (±0.2), 0.34 (± 0.09) and 0.03 (±0.21) of overall gene expression in case #7, case #8 and controls, respectively. The ∆17,18 transcript is undetectable in both variant cases and controls. M, molecular weight marker (0.15–2.1 Kbp, Roche and ΦX174 DNA/BsuRI (HaeIII) Marker, Thermo Scientific); C, healthy control sample; NTC, no template control.

**Figure 3:**
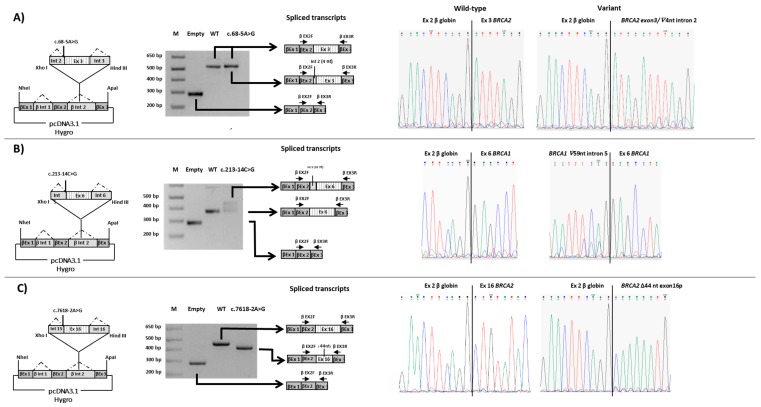
RT-PCR analysis of the *BRCA1* and *BRCA2* minigene constructs expressed in HeLa cells using vector specific primers. A schematic representation of the hybrid minigene constructs is depicted on the left; the dotted lines indicate normal splicing. Each construct contains the exon close to, or harboring the variant of interest and part of the upstream and downstream introns of *BRCA1* or *BRCA2* genes. Electropherograms of the spliced transcripts are shown on the right. (**A**) The construct contains exon 3 of the *BRCA2* gene. c.68-5A>G, construct containing the c.68-5A>G variant. (**B**) The construct contains exon 6 of the *BRCA1* gene. c.213-14C>G, construct containing the c.213-14C>Gvariant. (**C**) The construct contains exon 16 of the *BRCA2* gene. c.7618-2A>G, construct containing the c.7618-2A>G variant. M, 1 kb plus molecular marker (Invitrogen); Empty, empty β-globin-pCDNA3.1 vector; WT, wild-type construct.

**Figure 4:**
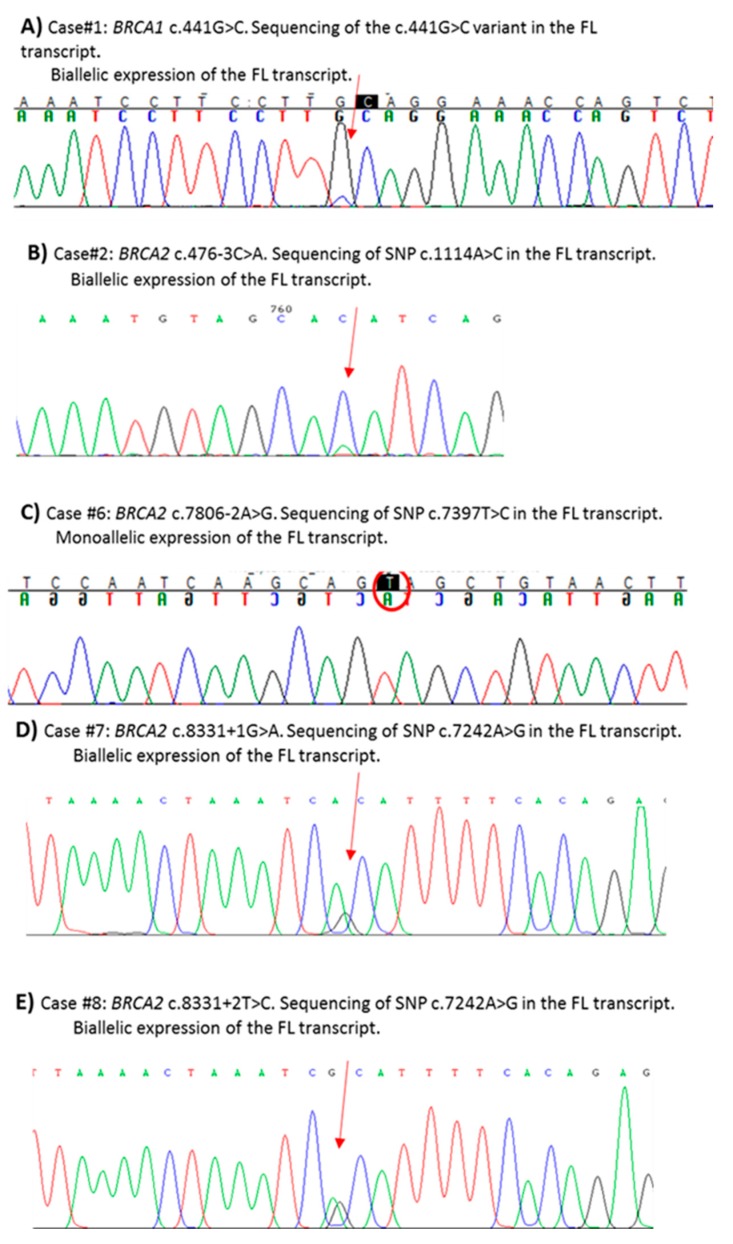
Determination of partial/total effect by SNP Sanger sequencing in the full-length (FL) transcripts from PAXgene samples (panel **A**) or LCLs (panels **B**–**E**). Primers are shown in Supplementary Table S3. The arrows indicate the investigated SNPs.

In the present study, we assessed the pathogenicity of ten unclassified variants in *BRCA1/2* identified in index cases selected from families undergoing genetic counseling and genetic testing. Nine variants were intronic (three in *BRCA1* and six in *BRCA2*) and one was located at the last nucleotide of *BRCA1* exon 7. By patient mRNA analysis, aberrant splicing patterns were demonstrated for eight variants (Figure 1 and Figure 2 and Table 1). Different types of abnormalities were observed: (1) alternative usage of canonical splice sites, including whole intron retention (c.441G>C in *BRCA1*) (Figure 1A) and whole exon skipping (c.476-3C>A in *BRCA2*) (Figure 2A); (2) usage of de novo splice sites (c.68-5A>G in *BRCA2*) (Figure 2B and Figure 3A) and usage of cryptic splice sites (c.213-14C>G in *BRCA1* and c.7618-2A>G, c.7806-2A>G, c.8331+1G>A and c.8331+2T>C in *BRCA2*) (Figure 1B and Figure 2C–E). In all cases, variant alleles produced transcripts leading to PTCs or in-frame deletions disrupting clinically important domains. Quantitative imbalances between physiological isoforms and aberrant transcripts were assessed by qPCR assays (Figure 1 and Figure 2). Complete/partial effect on splicing was established by the use of tag SNPs, whenever possible, or, alternatively, by monoallelic minigene assays, leading to variant classification according to current guidelines (Figure 3 and Figure 4) [37]. Consistent with in silico predictions, for two variants (c.302-5T>C and c.441+5A>G in *BRCA1*) a spliceogenic effect was not observed following patient mRNA analysis combined with methods preventing nonsense-mediated decay (NMD), or assessing allele-specific expression by tag-SNP analysis (Supplementary Figure S1).

### 2.1. Alternative Usage of Canonical Splicing Sites

One variant in *BRCA1* (c.441G>C), affecting the last nucleotide of exon 7, and for which conflicting interpretations of pathogenicity are reported in ClinVar (Table 1), was predicted by two out of five in silico analyses to damage the donor splice site (Table 2). Firstly, the hypothesis of exon 7 skipping was tested, but the detection of no alteration led us to postulate an intronic retention. The RT-PCR experiment for intronic retention showed the presence of an aberrant splicing product consisting of the whole intron 7 exonization, predicted to result in an early truncated protein lacking most functional domains (p.(Gln148Valfs*21)) (Figure 1A). qPCR quantified a contribution of full-length and aberrant transcript to the total *BRCA1* expression of 66% and 34%, respectively. No intron 7-retaining transcript was found in controls (Figure 1A). The use of the exonic variant itself (c.441G>C) as tag-SNP analysis allowed for the determination of a partial splicing effect (Figure 4A).

The c.476-3C>A variant in *BRCA2*, occurring at the 3’ splice site of intron 5 and reported with conflicting interpretations of pathogenicity in ClinVar (Table 1), was predicted to impair the canonical acceptor site by all bioinformatic tools (Table 2). Analysis of patient’s RNA showed that the variant leads to the increase of two naturally occurring isoforms with different out-of-frame deletions: one lacking exon 6 (∆6) and the other lacking both exons 5 and 6 (∆5,6) (Figure 2A) [38]. Both transcripts result in early truncated proteins, defective of all functional domains. By qPCR all isoforms were quantified in patient sample and controls. Quantitative analysis showed that both alternative isoforms (∆6 and ∆5,6), barely detectable in controls, were highly expressed in patient sample, at levels comparable with the full-length transcript (Figure 2A). Using an exonic tag SNP (c.1114A>C; p.(Asn372His)), we demonstrated that canonical transcript mostly derives from wild-type allele but a very small peak corresponding to the SNP variant associated with the mutant allele was observed, indicating a minimal “leaky” effect (Figure 4B).

### 2.2. Usage of A De Novo Splice Site

The c.68-5A>G substitution in *BRCA2*, reported as VUS in Clin Var, (Table 1), reduces the strength of the canonical 3′ splice site in intron 2 and simultaneously generates an alternative acceptor splice site four nucleotides upstream, predicted by four out of five in silico tools (SSF, MES, NNSPLICE and HSF) (Figure 2B and Table 2). The usage of this de novo splice site generates an isoform predicted to result in an early stop of translation with the consequently loss of all protein domains (p.(Leu24Argfs*7)) (Figure 2B). Simultaneously, the variant up-regulates the naturally occurring *BRCA2* isoform carrying the in-frame skipping of exon 3 (∆3) (Figure 2B). Expression quantification by qPCR revealed that the c.68-5A>G variant induces an increase of ∆3 isoform (7% of the total transcript comparable to the one induced by the c.68-7T>A variant found in another patient of our cohort (8% of the total transcript) vs. 2% in controls (mean value of ten samples) (Figure 2B). Minigene assay confirmed the generation of the out-of-frame isoform with the inclusion of four intronic nucleotides, but failed to detect the ∆3 isoform possibly due to the short sequence included in the construct that contains exon 3 and partial intronic flanking regions. The latter might not be enough to allow an alternative splicing mechanism. Sequencing of minigene product showed a very low level of full-length transcript produced from the mutated allele (Figure 3A and Table 1).

### 2.3. Usage of Cryptic Splice Sites

The c.213-14C>G variant in *BRCA1*, reported as VUS in ClinVar (Table 1), was predicted by all Alamut tools to activate a cryptic 3′ splice site at position c.213-59. In addition, three out of five interrogated tools (SSF, MES and HSF) predicted the creation of a de novo 3′ splice site at c.213-13 with a lower score respect to the cryptic site (Table 2). In vitro analyses revealed an abnormal product consisting of the out-of-frame retention of 59 bases at the 3′ end of intron 5 (Figure 1B). This partial intronic retention creates a premature stop codon with loss of all functional domains (p.(Arg71Serfs*11)). qPCR showed a low relative decrease of the full-length transcript in patient compared to control samples (87% vs. 100%) and that the aberrant product, totally absent in controls, contributed for 13% of the total *BRCA1* expression (Figure 1B). Hybrid minigene assay displayed a partial spliceongenic effect (Figure 3B). No effect due to the predicted creation of an alternative splice site at c.213-13 was observed.

The c.7618-2A>G variant in *BRCA2*, reported as pathogenic or likely pathogenic in ClinVar (Table 1), was predicted by all in silico tools to abolish the canonical 3′ splice site (Table 2). By RT-PCR we demonstrated the skipping of 44 nt at the 5′-end of exon 16 due to the activation of an exonic cryptic acceptor site (Figure 2C) predicted by only two (MES and HSF) out of five in silico tools (Table 2). The predicted effect on the protein was the disruption of the DNA-binding domain (DBD) (p.(Leu2540Glnfs*11)). qPCR demonstrated a low contribution (10%) of the mutant transcript (∆44nt-ex16) to the total gene expression (Figure 2C). Minigene assay confirmed the splicing pattern observed in patient’s mRNA and demonstrated a total spliceogenic effect (Figure 3C).

The *BRCA2* c.7806-2A>G variant, pathogenic or likely pathogenic in ClinVar (Table 1), was strongly predicted by all the in silico tools to abolish the natural acceptor splice site. In addition, the activation of two cryptic splice sites at positions c.7826 and c.7875 was predicted (Table 2). The in vitro analysis confirmed a complex abnormal splicing pattern leading to the production of three different isoforms which include the whole deletion of exon 17 (∆17), and the partial deletion of either 20 or 69 nucleotides at the 5′-end of exon 17 (∆20nt-ex17 and ∆69nt-ex17) (Figure 2D). All alternative transcripts lead to the disruption of the DBD domain. The relative amount of each transcript on the total *BRCA2* expression was investigated by qPCR. The ∆20nt-ex17 transcript accounted for 10% of overall expression in the case sample and is quite absent in controls, while ∆69nt-ex17 was barely detected in the case and totally absent in controls. Quantification of ∆17 was not possible by qPCR, in both the case and controls, probably due to the very low expression levels. The analysis of the c.7397 tag SNP in exon 14, showing mono-allelic expression of the full-length transcript, proved a total spliceogenic effect of the variant considered (Figure 4C).

The two alterations affecting the invariant dinucleotides at the 5′ end of intron 18 of *BRCA2*, c.8331+1G>A and c.8331+2T>C, were predicted by five and four in silico tools, respectively, to abolish the corresponding natural donor splice site (Table 2). Both variants are classified as pathogenic or likely pathogenic in ClinVar (Table 1). The in vitro characterization confirmed in both cases the same abnormal splicing pattern consisting in the skipping of the last 151 bp of exon 17 and of the whole exon 18 (∆151nt_ex17 + ∆18), in addition to the increase of the two naturally occurring isoforms involving exon 18 (∆18 and ∆17,18) (Figure 2E) [38]. Quantification by qPCR of all detected transcripts revealed the relative decrease of the full length *BRCA2* mRNA in patient samples (67% for c.8331 + 1G > A and 61% for c.8331+2T>C vs. 96% in healthy control samples). The ∆18 isoform was found up-regulated in both samples (23% and 34% in c.8331+1G>A and c.8331+2T>C, respectively, vs. 3% in controls), while the lowly expressed ∆17,18 isoform could not be quantified both in samples and controls. The aberrant transcript lacking the last 151 bp of exon 17 and the entire exon 18, not expressed in controls, was shown to contribute for 9% and 4% of the total expression in c.8331+1G>A and c.8331+2T>C carriers, respectively. The analysis of the c.7242A>G tag SNP in exon 14 demonstrated a bi-allelic expression of the full-length transcript in both cases (Figure 4D,E).

### 2.4. No Splicing Effect

For two variants in *BRCA1*, namely c.302-5T>C and c.441+5A>G, located in the 3′ and 5′ Cartegni consensus regions of intron 6 and intron 7, respectively, we did not detect any splicing aberration, as expected by the in silico prediction (Supplementary Figure S1).

For the c.441+5A>G, the use of an exonic tag SNP (c.4308T > C) allowed to demonstrate biallelic expression (Supplementary Figure S1B). No exonic tag SNPs were available for the c.302-5T>C variant. Analysis of minigene expressed in cells treated with puromycin to prevent potential degradation of unstable transcripts via NMD [33], confirmed the presence of only the full-length transcript (Supplementary Figure S1A).

## 3. Discussion

*BRCA1* and *BRCA2* genetic test is fundamental for counseling and clinical management of high-risk breast-ovarian families. Massive parallel sequencing has greatly accelerated the analysis and the diagnostic process is generally straightforward for protein-truncating mutations. However, the interpretation of the biological consequences of the other types of variants still represents a challenge, creating problems in the clinical setting. Given that a significant fraction of VUS can have spliceogenic effects, transcript analysis represents an important tool to assess pathogenicity. Here, by patients′ mRNA analysis, we characterized the impact on splicing of ten *BRCA1/2* variants. For all these variants, multifactorial likelihood analysis was not applied due to the lack of sufficient data. Eight variants were demonstrated to impact on the normal splicing process and aberrant transcripts were characterized. Different types of abnormal splicing events were observed, including intron retention (c.441G>Cin *BRCA1*), exon skipping (c.476-3C>A in *BRCA2*), and alternative 5′ or 3′ splice site activation (c.213-14C>G in *BRCA1* and c.68-5A>G, c.7618-2A>G, c.7806-2A>G c.8331+1G>A, c.8331+2T>C in *BRCA2*), leading to transcripts with PTCs or in-frame loss of functionally important residues. Partial/complete splicing impact and quantitative contribution of different transcripts were assessed leading to variants classification according to current guidelines developed by the ENIGMA consortium [37].

We demonstrated that the c.441G>C variant in *BRCA1* causes the retention of intron 7. In the absence of in vitro studies assessing allele-specific transcript expression, this variant, causing G > non-G substitution at the last base of the exon and with the first 6 bases of the intron not being GTRRGT, would have been considered of Class 4 (likely pathogenic) [37]. In the present study, the usage of the variant itself as a tag-SNP allowed to reveal a partial splicing effect, leading to a Class 3 (VUS) reassignment, in accordance with ENIGMA guidelines [37]. As to the c.476-3C>A variant in *BRCA2*, we observed the up-regulation of transcripts carrying the out-of-frame deletions of exon 6 (∆6) and exons 5–6 (Δ5–6). Both isoforms were barely detectable in control samples, in accordance with previous studies reporting these exon-skipping events as naturally occurring at low expression levels [21,33,38]. Up-regulation of these non-functional products had been previously reported in association with the c.476-2A>G, c.516G>A and c.516+1G>T pathogenic *BRCA2* variants [28,33,39]. By real-time qPCR, we demonstrated that the c.476-3C>A variant causes the up-regulation of both alternative isoforms at a level comparable with that of the full-length transcript. Since a minimal “leaky” effect was demonstrated by the use of an exonic tag SNP, current guidelines indicate to consider the variant of Class 3 and recommend multifactorial likelihood analysis to assess a definitive pathogenic role [37].

Concerning the activation of alternative splice sites, we found that the c.68-5A>G variant in *BRCA2*, creating a de novo acceptor splice site four nucleotides upstream exon 3, leads to the production of an out-of-frame transcript predicted to result in an early interruption of translation (p.(Leu24Argfs*7)). Simultaneously, we observed an up-regulation of a naturally occurring *BRCA2* isoform carrying the in-frame skipping of exon 3 (∆3). In accordance with previous data [38,39,40], the ∆3 isoform was present in our control samples, representing a non-negligible fraction of the total transcript. The extent of up-regulation of the ∆3 isoform in the presence of the c.68-5A > G variant (approximately 7%) was comparable with that found in one patient carrying the already reported c.68-7T>A *BRCA2* variant (approximately 8%). The c.68-7T>A substitution has been the object of intense debate concerning its pathogenetic role [14,19,27,40,41], but a recent article finally classified it as benign [42]. The c.68-5A>G variant here identified, with respect to the c.68-7T>A substitution, generated an additional transcript containing an early PTC. The up-regulation of the in-frame alternative ∆3 isoform may raise concerns about the clinical impact of the variant. However, exclusive expression of the ∆3-transcript from one allele has been demonstrated to be associated with a high risk of breast and/or ovarian cancer [40,41]. In fact, residues encoded by exon 3 have been demonstrated to be important for the interaction with PALB2, a cofactor essential for the nuclear localization of the BRCA2 protein, the interaction with BRCA1, the recruitment of RAD51 to the DNA double strand breaks (DSBs), and the protection of the replication forks. In fact, the disruption of the interaction between PALB2 and BRCA2 has been demonstrated to impair the DNA DSBs repair function [41,43,44,45,46]. Moreover, the results from multifactorial likelihood analysis of six *BRCA2* variants leading to full exon 3 skipping strongly inclines in favor of a pathogenic role of these variants [41]. Notwithstanding, minigene analysis revealed the expression the of full-length transcript from the mutated allele, although at very low level, which, formally does not allow to classify the c.68-5A > G variant as pathogenic, in the absence of additional evidences.

Activation of cryptic splice sites was demonstrated for the remaining five spliceogenic variants (c.213-14C>G in *BRCA1* and c.7618-2A>G c.7806-2A>G c.8331+1G>A, c.8331+2T>C in *BRCA2*). The c.213-14C>G variant in *BRCA1* was predicted to weaken canonical splicing and to activate a cryptic 3′ splice site. Patient mRNA analysis confirmed the prediction and showed the out-of-frame retention of 59 bp at the 3′ end of intron 5. The usage of the same cryptic acceptor site had been previously reported for the c.213-11T>G pathogenic variant, suggesting that this site is preferentially used when the canonical one is weakened or eliminated [33]. The exonization of this part of intron 5 is associated with a very low expression level of the resulting transcript, compared to full-length, as shown by quantitative expression analysis. This is consistent with the evidence of the partial splicing effect detected by minigene assay and supports the classification of the variant as Class 3 [37]. The c.7618-2A>G variant in *BRCA2*, predicted by all in silico tools to abolish the canonical 3′ splice site, showed an aberrant out-of-frame transcript devoid of the first 44 nt of exon 16, due to the activation of a cryptic exonic splice site predicted by only two out of five prediction tools (MES and HSF). The contribution of the aberrant transcript to the total gene expression was very low (approximately 10%) suggesting a possible role of NMD in triggering the degradation of the mutant transcript in patient’s cell lines. However, this possibility was not experimentally verified by puromycin treatment. The same aberration had been previously demonstrated for the adjacent c.7618-1G>A pathogenic variant [28]. Since no informative (heterozygous) *BRCA2* exonic SNPs were present in carrier of the c.7618-2A>G, allele-specific expression was assessed by minigene assay, which represents a valuable tool to guide clinical classification of spliceogenic variant in cases where tag-SNP analyses cannot be performed. The assay showed the production of only the mutant transcript, allowing the classification of the variant as pathogenic. The c.7806-2A>G variant in *BRCA2*, predicted by all the in silico tools to abolish the canonical 3′ splice site, in addition to the skipping of exon 17, leads to the activation of two exonic cryptic splice sites, producing two transcripts lacking respectively 20 and 69 nt at the 5′-end of exon 17. All the produced mRNAs carry a PTC leading to the loss of the DBD. Noticeably, in a previous investigation of the variant, only the transcript missing 20 nt was observed, whereas the two transcripts lacking the entire exon 17 and the 69 nt at the 5′-end of the exon were not detected [19]. The ratio of the total amount of the aberrant transcripts, taken together, to the total *BRCA2* expression was very low (~10%). Given the exclusion of a leaky effect of the mutation by tag-SNP analysis, we can hypothesize a massive degradation of the aberrant transcripts possibly induced by the formation of PTCs. However, also for this sample puromycin treatment was not performed. These findings, obtained by a direct patient’s mRNA analysis, are concordant with the data obtained with the minigene assay by Fraile-Bethencourt et al. [36], and contribute to the final classification of the described variant as pathogenic, given that no full-length transcript was found to be produced by the mutated allele. The two variants c.8331+1G>A and c.8331+2T>C, both predicted to abolish the canonical exon 18 donor splice site, generate three mis-spliced transcripts, each carrying a PTC. Two of these transcripts (∆18 and ∆17,18) are reported as naturally occurring variants, found in normal samples at low levels [38]. Quantitative analysis showed a relative increase of ∆18 isoform in cases respect to controls, while neither in cases nor in controls the amount of the ∆17,18 isoform was detected by qPCR, probably due to technique limitations. The third transcript, showing the loss of 151 bp at exon 17 3′-end and of exon 18 (∆151nt_ex17 + ∆18), was generated by the usage of an exonic cryptic splice site, probably activated following the loss of the canonical donor site of exon 18. This transcript is exclusively expressed in cases and its contribution to the overall *BRCA2* expression is higher in the c.8331+1G>A carrier (9%) than in the c.8331+2T>C carrier (4%). The alternative transcripts overall contribute to the total *BRCA2* expression for 32% and 38% in c.8331+1G>A and c.8331+2T>C, respectively. These two variants affect the same splice site of the c.8331+1G>T, previously reported to induce aberrant splicing and classified as pathogenic by Fraile-Bethencourt et al. [36]. In the same study, these authors classified as pathogenic also the c.8331+2T>C, based on the observation, by minigene assay, of no full-length expression by the mutated allele. By contrast, in our study, tag-SNP analysis revealed the expression of the normal transcript from both the wild-type and the mutated allele. While this discrepancy could be attributed to the different approaches used in the two studies to assess allele-specific expression, our results argue against a classification of the c.8331+2T>C as pathogenic (Class 5). The same applies also to the c.8331+1G>A, which in our hands displays a bi-allelic full-length expression. Therefore, in our opinion, in the absence of further evidences, both the above variants should be classified at present as “uncertain” (Class 3), according to the ENIGMA guidelines [37].

This study allowed the assessed for the first time the complete aberrant splicing effect in patients’ mRNA for the c.7618-2A>G and c.7806-2A>G *BRCA2* variants, providing strong evidence of their pathogenicity. These findings have important consequences for families in terms of preventive and prophylactic measures, as well as targeted therapies such as those based on the use of PARP-inhibitors. In accordance with in silico prediction, we demonstrated that the c.441+5A>G and c.302-5T>C variants in *BRCA1* have no splicing impact, allowing a final classification as likely benign [37]. Finally, due to evidences of a partial splicing effect, six variants (c.213-14C>G, c.441G>C in *BRCA1* and c.68-5A>G, c.476-3C>A, 8331+1G>A and 8331+2T>C in *BRCA2*) remain of Class 3 in accordance with current interpretation guidelines [37]. This conservative criterion is justified considering that spliceogenic variants preserving the ability to synthesize a normal (full-length) transcript do not completely abrogate the synthesis of a normal protein and might maintain a tumor suppressor activity [25]. At least in some instances, these alterations might be associated with a lower tumor risk, compared to classical pathogenic BRCA gene variants. This justifies caution in patients’ management although, if present in patients with ovarian cancer, they could anyhow be targeted by PARP inhibitors, in view of the potential beneficial effect of such therapy.

## 4. Patients and Methods

### 4.1. Patients

Patients were selected among those who attended three different Italian centers (Azienda Ospedaliera Universitaria Senese, Fondazione IRCCS, Istituto Nazionale dei Tumori (INT) of Milan, Azienda Ospedaliero-Universitaria di Modena) for genetic counseling and *BRCA1/BRCA2* testing. Genetic analysis was offered based on individual and/or family history according to updated National Comprehensive Cancer Network (NCCN) guidelines (NCCN Clinical Practice Guidelines in Oncology: Genetic/Familial High-Risk Assessment: Breast and Ovarian) or local inclusion criteria [47]. Sporadic cases of OC accessed to the molecular analysis on the basis of national recommendations [48]. After a first DNA-level analysis performed by massive parallel sequencing (Ion Torrent PGM, Life Technologies, Carlsbad, CA, USA), or conventional Sanger sequencing, patients with VUS that could potentially alter the mRNA splicing process were selected for in vitro studies. The significance and consequences of VUS identification were discussed with patients in post-test counseling. New blood samples were thus collected in order to investigate the pathogenicity of the variants by transcript analysis. Informed consent was obtained prior to testing and the study was approved 28 January 2015 by the local Ethics Committee of the Istituto Nazionale Tumori (INT); ethic code: N. INT 7/15.

### 4.2. Methods

#### 4.2.1. In Silico Splicing Analysis

Five tools integrated in the Alamut Visual software application (Interactive Biosoftware, Version 2.7, Rouen, France) were used for in silico prediction of the impact on the splicing process: Splice Site Finder-like (SSF), MaxEntScan (MES) Splice Site Prediction by Neural Network (NNSPLICE) GeneSplicer (GS) and Human Splicing Finder (HSF) [16,49,50,51,52,53].

#### 4.2.2. Cell Cultures

Mononuclear cells were isolated from 5 mL of peripheral blood supplemented with Ficoll. Epstein-Barr virus (EBV)-immortalized human lymphoblastoid cell lines (LCLs) were established and grown as previously reported [33].

#### 4.2.3. RNA Extraction and Reverse Transcriptase-PCR (RT-PCR)

Depending on the strategies used in the participating laboratories, different protocols of RNA extraction from blood were used. Total RNA was isolated from PAXgene blood RNA tubes (PreAnalytiX®, Qiagen, Hilden, Germany) (http:/www.qiagen.com), with PAXgene Blood RNA Kit (IVD) (PreAnalytiX®), following the manufacturer’s instructions and quantified by NanoDrop 2000 Spectrophotometer (Thermo Scientific). For each sample, 1 µg of RNA was reverse-transcribed into cDNA using a dedicated Qiagen kit (QuantiTect® Reverse Transcription Kit, Qiagen) according to the manufacturer’s instructions. RNA from LCLs was extracted and reversed transcribed as described [42]. cDNA was stored at −20 °C until use.

#### 4.2.4. Transcript Characterization

In order to verify the effect of considered VUS on BRCA gene splicing, specific PCR primers were designed (Supplementary Table S1). For each amplification, a free-cDNA sample and five normal control cDNA samples were included. PCRs were performed using GoTaq DNA Polymerase reagents (5× GoTaq Flexi buffer; 25 mM MgCl_2_, GoTaq Hot Start Polymerase), in addition with 2 mM dNTPS, milliQ H_2_O and 100 ng of cDNA, for a final volume of 50 µL. Amplification was performed in a Thermal Cycler 2720 (Applied Biosystem, Foster City, CA, USA) with the following PCR program: 95 °C for 5 min; 35 cycles at 95 °C for 30 s, at the specific annealing temperature for 30 s, at 72 °C for 60 s; 72 °C for 5 min. Then, 10 µL of amplified PCR products were mixed with the loading dye, containing Bromophenol Blue, and separated by electrophoresis on a 3% agarose gel. Bands were displayed in an UV transilluminator. In all cases in which it was possible to discriminate different gel bands, the different amplification products were purified using the Zyomoclean Gel DNA Recovery kit (Zymo research, Irvine, CA-USA). In the remaining cases, unfractionated PCR products were purified and cloned as previously described [33]. Each cDNA PCR products were sequenced using PE Big dye terminator cycle sequencing kit on an ABI Prism 3130 Genetic Analyzer (Applied Biosystems). The software Sequencer (v.4.9) was used for sequence analysis.

#### 4.2.5. WT/Mutant Transcript Quantification by Quantitative Polymerase Chain Reaction (qPCR)

To quantify the relative expression levels of transcripts in mutant and control samples, quantitative polymerase chain reaction (qPCR) with isoforms-specific primers (Supplementary Table S2) was performed. A region not involved in splicing aberrations, and not subject to naturally occurring alternative splicing [38,54], was selected as internal control to take into account the inter-individual variability of *BRCA1*/*2* expression. *GUSB* was used as housekeeping gene to normalize cDNA amounts [42]. For controls the average value of ten samples was considered. Quantitative PCR was carried out in single-plex reactions in a 96-well optical plate with FastStart SYBR Green Master Mix (Roche) on the ABI Prism 7000 Sequence Detection System (Applied Biosystems). Experiments were performed in triplicate in a final volume of 20 µL with 25 ng of cDNA and 25 nM of each primer, following the SYBR Green protocol (Roche, Basel, Switzerland). Standard thermal cycling conditions were: 2 min at 50 °C and 10 min at 95 °C; 40 cycles at 95 °C for 15 s and 60 °C for 1 min; dissociation stage 95 °C for 15 s, 60 °C for 20 s and 95 °C for 15 s. The mean value of each triplicate was firstly normalized to the housekeeping gene (∆Ct). The ∆Ct of the full-length and aberrant transcripts was then normalized to that of the internal control (∆∆Ct), applying the comparative Ct method to obtain the relative expression quantification [55]. In each sample, the expression levels of all observed fragments were expressed as a percentage of the total expression of all fragments, i.e., analyzed transcript/(full-length + alternative transcripts).

#### 4.2.6. Assessment of Partial/Total Effect on Splicing by the Use of Exonic Tag SNPs

Whenever possible informative exonic SNPs were analyzed to assess a total or partial effect on splicing of investigated intronic variants. For each variant a PCR fragment spanning the exonic tag-SNP was amplified from the canonical (full-length) transcript by the use of specific primers (Supplementary Table S3) and sequenced to assess mono or biallelic expression.

#### 4.2.7. In Vivo Splicing Assay with Minigenes

A portion of the gene containing the exon close to or harboring each variant of interest and part of the flanking introns was amplified from patients’ genomic DNA (oligonucleotides and PCR conditions are available upon request). PCR fragments were subsequently cloned into the β-globin-pCDNA3.1 vector to produce the hybrid minigene constructs, as previously described [56,57]. The mutant minigene constructs, their corresponding wild-type versions or the empty vector were transfected into HeLa cells and after 24 hours total RNA was extracted using the TRIzol kit (Invitrogen) and reverse transcribed. The cDNA was then amplified using primers located on β-globin exons 2 and 3 and PCR fragments were separately sequenced as previously reported [57]. Puromycin treatment of cell line transfected with the minigene carrying the *BRCA1* c.302-5T > C variant was performed as described [33].

## 5. Conclusions

Our results provide further evidence of the usefulness of mRNA analysis for the clinical classification of variants in disease-associated genes. In fact, through this approach we were able to provide evidence assessing two *BRCA2* variants (c.7618-2A>G and c.7806-2A>G) as pathogenic and two *BRCA1* variants (c.302-5T>C and c.441+5A>G) as benign. However, we also observed that a large fraction (*n* = 6) of investigated spliceogenic variants retain the ability to produce (although at variable levels) functional full-length transcripts, and therefore, cannot be definitively classified as pathogenic according to current guidelines.

Due the availability of specific risk-reduction measures and new therapeutic approaches for carriers of pathogenic BRCA gene variants, the development of ad hoc strategies for the classification of variants with a partial splicing effect has become an urgent need. Along this line, two recent studies of the ENIGMA consortium, combining genetic and clinical data analysis with accurate mRNA isoform quantification, were able to demonstrate the non-pathogenicity of the *BRCA1* c.[594-2A>C; 641A>G] and *BRCA2* c.68-7T>A leaky splicing variants [42,58]. In both cases, these investigations defined the threshold for ratio between altered and functionally proficient gene transcripts not associated with high cancer risk. These observations could inform the classification of other BRCA gene variants causing partial spliceogenic effects, thus improving the informativeness of BRCA testing and the number of families and individuals that can benefit from such tests.

## Figures and Tables

**Table 1:** mRNA splicing effects of identified variants and predicted effect on protein.

Gene	Variant (Human Genome Variant Society, HGVS) Chromosome Position (GRCh37)	Location	ClinVar Classification (Entries) *	Methods	Observed Effect on Splicing	Effect on mRNA (HGVS)	Effect on Protein (HGVS)	Complete/Partial Effect (Method)	Splice Class †	Variant Classification According to Published Guidelines ^§^
*BRCA1*	c.213-14C>G Chr17:g.41256987G>C	Intron 5	VUS (2)	PAXgene	retention of 59nt at 3′-end of intron 5	r.[212_213ins213-59_213-1; 213-14c > g]	p.Arg71Serfs*11	Partial (minigene)	2S	Class 3
	c.302-5T>C Chr17:g.41256283A > G	Intron 6	VUS (1)	PAXgene	no effect	NA	NA	NA	1S	Class 2
	c.441G>C Chr17:g.41256139C>G	Exon 7	LP (1)/VUS (3)	PAXgene	retention of intron 7	r.[441_442ins441+1_442-1; 441g > c]	p.Gln148Valfs*21	Partial (exonic tag SNP)	2S	Class 3
	c.441 + 5A>G Chr17:g.41256134T>C	Intron 7	NP	PAXgene	no effect	NA	NA	NA	1S	Class 2
*BRCA2*	c.68-5A>G Chr13:g.32893209A>G	Intron 2	VUS (1)	lymphocytes	retention of 4nt at 3′-end of intron 2; up-regulation of ∆3 isoform	r.[67_68ins68-4_68-1]; ↑ r.[68_316del]	p.[Leu24Argfs*7]; ↑ p.[Asp23_Leu105del]	Partial (minigene)	3S	Class 3
	c.476-3C>A Chr13:g.32900376C>A	Intron 5	P (1)/VUS (1)	LCL	up-regulation of ∆5,6 and ∆6 isoforms	↑ r.[426_516del]; ↑ r.[476_516del]	↑ p.[Ser142Argfs*13]; ↑p.[Val159Glyfs*10];	Partial (exonic tag SNP)	2S	Class 3
	c.7618-2A>G Chr13:g.32931877A>G	Intron 15	P (1)/LP (1)	PAXgene	skipping of 44nt at 5′-end of exon 16	r. [7618_7661del]	p.[Leu2540Glnfs*11]	Complete (minigene)	3S	Class 5
	c.7806-2A>G Chr13:g.32936658A>G	Intron 16	P (8)/LP (1)	LCL	skipping of 20nt at 5′-end of exon 17; skipping of 69nt at 5′-end of exon 17; skipping of exon 17	r.[7806_7825del]; r.[7806_7874del]; r. [7806_7976del]	p.[Ala2603Cysfs*8]; p.[Ala2603_Arg2625del]; p.[Ala2603Serfs*3]	Complete (exonic tag SNP)	3S	Class 5
	c.8331 + 1G > A Chr13:g.32937671G>A	Intron 18	P (3)/LP (1)	LCL	skipping of 151nt at 3′-end of exon 17 and skipping of ex18; up-regulation of ∆ex17,18 and ∆ex18 isoforms	r.[7826_8331del]; ↑ r.[7806_8331del]; ↑ r.[7977_8331del];	p.[Gly2609Aspfs*4]; ↑ p.[Ala2603Phefs*43]; ↑ p.[Tyr2660Phefs*43];	Partial (exonic tag SNP)	2S	3
	c.8331+2T>C Chr13:g.32937672T>C	Intron 18	P (5)/LP (1)	LCL	skipping of 151nt at 3′-end of exon 17 and skipping of ex18; up-regulation of ∆ex17,18 and ∆ex18 isoforms	r.[78268331del]; ↑ r.[7806_8331del]; ↑ r.[7977_8331del]	p.[Gly2609Aspfs*4]; ↑ p.[Ala2603Phefs*43]; ↑ p.[Tyr2660Phefs*43];	Partial (exonic tag SNP)	2S	3

* Clinvar (URL: https://www.ncbi.nlm.nih.gov/clinvar/; last update 26 January 2019). VUS, variants of uncertain significance; LP, likely pathogenic; NP not provided; P, pathogenic; LCL, lymphoblastoid cell line; ^†^ Class 1S, no effect on splicing; Class 2S, leaky splice site variant: partial effect; Class 3S, severe impact on splicing: the mutant variant does not produce naturally occurring functional transcripts [19]. ^§^ Class 5, pathogenic; Class 4, likely pathogenic; Class 3, uncertain; Class 2, likely not pathogenic or of little clinical significance; ^§^ according to [37]; NA, not applicable.

**Table 2:** In silico analyses for variants leading to the usage of cryptic/de novo splice sites.

Variant	Location, SS, Distance	Canonical SS					Used Cryptic SS (Location, SS)					De Novo SS				
		Wt > Mut (% Variation)					Score					Score				
		MES	SSF	NNSPLICE	GeneSplicer	HSF	MES	SSF	NNSPLICE	GeneSplicer	HSF	MES	SSF	NNSPLICE	GeneSplicer	HSF
Gene:*BRCA1* c.213-14C>G	intron 5, 3′, 14						c.213-59 (intron 5, 3′)					c.213-13 (intron 5, 3′)				
		4.84 > 2.23 (−53.9%)	85.51 > 83.10 (−2.8%)	NP	NP	=89.39	10.23	86.74	0.97	5.90	82.22	2.06	79.09	NP	NP	80.34
c.302-5T>C	Intron 6, 3′, 5	11.7 > 11.2 (−4.4%)	91.5 > 88 (−3.9%)	0.99 > 0.98 (−0.7%)	8.4 > 7.5 (−11%)	NP	None					None				
c.441G>C	exon 7, 5′, 1	3.2 > -	NP	NP	NP	78.8 > 67.8 (−14%)	None					None				
c.441+5A>G	Intron 7, 5′, 5	3.2 > 8.8 (+173.7%)	- >82.0	- >1.0	- >2.9	NP	None					None				
Gene:*BRCA2* c.68-5A>G	intron 2, 3′, 5	6.10 > 0.68 (−88.9%)	87.89> -	0.94 > 0.93 (−1.0%)	NP	80.63 > 80.56 (−0.1%)	None					c.68-4 (intron 2, 3′)				
												6.12	88.92	0.96	NP	96.85
c.476-3C>A	Intron 5, 3′, 3	10.49 > 6.02 (−42.6%)	88.73 > 78.77 (−11.2%)	0.91> -	2.02> -	91.61 > 82.22 (−10.2%)	None					None				
c.7618-2A>G	intron 15, 3′,						c.7662 (exon 16, 3′)					NP				
		7.11>-	86.07 > -	0.69 > -	3.17 > -	79.10 > -	1.53	NP	NP	NP	73.60					
c.7806-2A>G	intron 16, 3′, 2	8.33 > -	98.45 > -	0.95 > -	3.73 > -	91.03 > -	c.7826 (exon 17, 3′)					NP				
							5.95	78.60	NP	NP	87.97					
							c.7875 (exon 17, 3′)									
							5.03	72.60	0.54	NP	78.95					
c.8331+1G>A c.8331+2T>C	Intron 18, 5′, 1 Intron 18, 5′, 2	8.88 > -	87.54> -	0.96> -	2.27> -	86.88> -	c.7826 * (exon 17, 5′)					NP				
		8.88>	87.54 > 87.12 (−0.5)	0.96>	2.27>	86.88>	6.34	80.45	0.94	NP	88.10					

In silico analysis was performed using five prediction tools (Max Ent Scan, Splice Site Finder-like, NNSPLICE, GeneSplicer, Human Splicing Finder). -, SS abolished by the variant; NP, not predicted. Thresholds: MaxEnt, (0–12) for the 5′SS and (0–16) for the 3′SS, SSF (0–100), NNSPLICE (0–1), GeneSplicer (0–15), HSF (0–100). * This position is outside the range of prediction of Alamut Visual (199 bp), so data relative to this cryptic splice site were collected a posteriori, after the experimental analysis of the splicing pattern.

## Supplementary Materials

The following are available online at https://www.mdpi.com/2072-6694/11/3/295/s1, Figure S1: mRNA analysis of non-spliceogenic *BRCA1* variants from PAXgene samples, Table S1: Primers for mRNA splicing, Table S2: Primers used in Real-time qPCR, Table S3: Primers used for Tag-SNPs sequencing.
